# Pharmacotherapy for behavioural manifestations in frontotemporal dementia: An expert consensus from the European Reference Network for Rare Neurological Diseases (ERN‐RND)

**DOI:** 10.1111/ene.16446

**Published:** 2024-10-24

**Authors:** Casper Wittebrood, Marina Boban, Annchiara Cagnin, Sabina Capellari, François‐Laurent De Winter, Atbin Djamshidian, Manuel Menéndez González, Lena E. Hjermind, Lenka Krajcovicova, Johanna Krüger, Johannes Levin, Kathrin Reetz, Eloy Rodriguez Rodriguez, Jonathan Rohrer, Tim Van Langenhove, Carola Reinhard, Holm Graessner, Robert Rusina, Dario Saracino, Marion Houot, Harro Seelar, Rik Vandenberghe

**Affiliations:** ^1^ Department of Neurology University Hospital Leuven Leuven Belgium; ^2^ Department of Cognitive Neurology, Referral Centre for Cognitive Neurology and Neurophysiology University Hospital Centre Zagreb Zagreb Croatia; ^3^ School of Medicine University of Zagreb Zagreb Croatia; ^4^ Department of Neuroscience and Padua Neuroscience Centre University of Padua Padua Italy; ^5^ Department of Biomedical and Neuromotor Science University of Bologna Bologna Italy; ^6^ IRCCS Istituto delle Scienze Neurologiche di Bologna Bologna Italy; ^7^ Department of Neurology Medical University Innsbruck Innsbruck Tyrol Austria; ^8^ Department of Neurology Hospital Universitario Central de Asturias Oviedo Spain; ^9^ Department of Medicine Universidad de Oviedo Oviedo Spain; ^10^ Instituto de Investigación Sanitaria del Principado de Asturias Oviedo Spain; ^11^ Department of Neuorology, Neurogenetics Clinic and Clinical Trial Unit, Danish Dementia Research Centre Copenhagen University Hospital, Rigshospitalet Copenhagen Denmark; ^12^ First Department of Neurology, St Anne´s University Hospital and Faculty of Medicine Masaryk University Brno Czech Republic; ^13^ Department of Neurology Neurocentre, Oulu University Hospital Oulu Finland; ^14^ Research Unit of Clinical Medicine University of Oulu Oulu Finland; ^15^ MRC, Oulu University Hospital Oulu Finland; ^16^ Department of Neurology LMU University Hospital, LMU Munich Munich Germany; ^17^ German Centre for Neurodegenerative Diseases Munich Germany; ^18^ Munich Cluster for Systems Neurology (SyNergy) Munich Germany; ^19^ Department of Neurology RWTH Aachen University Aachen Germany; ^20^ Neurology Service Marqués de Valdecilla University Hospital, Institute for Research Marqués de Valdecilla (IDIVAL) Santander Cantabria Spain; ^21^ CIBERNED, Network Centre for Biomedical Research in Neurodegenerative Diseases National Institute of Health Carlos III Madrid Spain; ^22^ Medicine and Psychiatry Department University of Cantabria Santander Spain; ^23^ Department of Neurodegenerative Disease, Dementia Research Centre UCL Institute of Neurology London UK; ^24^ Department of Neurology, Cognitive Centre Ghent University Hospital Ghent Belgium; ^25^ Centre for Rare Diseases and Institute of Medical Genetics and Applied Genomics University Hospital Tübingen Tübingen Germany; ^26^ Institute for Medical Genetics and Applied Genomics University of Tübingen Tübingen Germany; ^27^ Centre for Rare Diseases University Hospital Tübingen Tübingen Germany; ^28^ Department of Neurology, Third Faculty of Medicine Charles University and Thomayer University Hospital Prague Czech Republic; ^29^ Paris Brain Institute, Institut du Cerveau‐ICM, Inserm U1127, CNRS UMR 7225 AP‐HP‐Hôpital Pitié‐Salpêtrière, Sorbonne Université Paris France; ^30^ Reference Centre for Rare or Early Dementias, IM2A, Département de Neurologie AP‐HP‐Hôpital Pitié‐Salpêtrière Paris France; ^31^ Centre of Excellence of Neurodegenerative Disease (CoEN) AP‐HP, Pitié‐Salpêtrière Hospital Paris France; ^32^ Department of Neurology, Institute of Memory and Alzheimer's Disease (IM2A) AP‐HP, Pitié‐Salpêtrière Hospital Paris France; ^33^ Clinical Investigation Centre for Neurosciences Institut du Cerveau (ICM), Pitié‐Salpêtrière Hospital Paris France; ^34^ Department of Neurology and Alzheimer Centre Erasmus MC Erasmus MC University Medical Centre Rotterdam The Netherlands

**Keywords:** drug therapy, expert testimony, frontotemporal dementia, neurobehavioural manifestations, neurodegenerative diseases

## Abstract

**Background and Purpose:**

Frontotemporal dementia (FTD) is a neurodegenerative disorder characterized by pervasive personality and behavioural disturbances with severe impact on patients and caregivers. In current clinical practice, treatment is based on nonpharmacological and pharmacological approaches. Unfortunately, trial‐based evidence supporting symptomatic pharmacological treatment for the behavioural disturbances in FTD is scarce despite the significant burden this poses on the patients and caregivers.

**Method:**

The study examined drug management decisions for several behavioural disturbances in patients with FTD by 21 experts across European expert centres affiliated with the European Reference Network for Rare Neurological Diseases (ERN‐RND).

**Results:**

The study revealed the highest consensus on drug treatments for physical and verbal aggression, impulsivity and obsessive delusions. Antipsychotics (primarily quetiapine) were recommended for behaviours posing safety risks to both patients and caregivers (aggression, self‐injury and self‐harm) and nightly unrest. Selective serotonin reuptake inhibitors were recommended for perseverative somatic complaints, rigidity of thought, hyperphagia, loss of empathy and for impulsivity. Trazodone was specifically recommended for motor unrest, mirtazapine for nightly unrest, and bupropion and methylphenidate for apathy. Additionally, bupropion was strongly advised against in 10 out of the 14 behavioural symptoms, emphasizing a clear recommendation against its use in the majority of cases.

**Conclusions:**

The survey data can provide expert guidance that is helpful for healthcare professionals involved in the treatment of behavioural symptoms. Additionally, they offer insights that may inform prioritization and design of therapeutic studies, particularly for existing drugs targeting behavioural disturbances in FTD.

## INTRODUCTION

Frontotemporal dementia (FTD) encompasses a spectrum of clinical syndromes characterized by frontal and temporal atrophy, manifesting as behavioural, personality and language changes. Frontotemporal lobar degeneration pertains to the underlying neurodegenerative pathological changes in FTD syndromes. Although there are a number of ongoing trials [1], at present there are neither proven nor US Food and Drug Administration or European Medicines Agency approved disease‐modifying treatments for FTD. Therefore, the current therapeutic approach is purely symptomatic relying on a combination of nonpharmacological and off‐label pharmacological approaches lacking quality evidence of effectiveness.

Pharmacological treatment has been primarily focused on common neuropsychiatric symptoms in FTD, with less emphasis on executive dysfunction and working memory deficits. Selective serotonin reuptake inhibitors (SSRIs) are often used to treat FTD patients due to the established association between FTD and presynaptic serotonin deficit, alongside a loss of cortical serotoninergic innervation [2]. This pathophysiological basis for SSRI use is further supported by the favourable response to SSRIs of similar behavioural symptoms in patients with psychiatric disorders. Positive effects in FTD have been demonstrated in some small open‐label trials or case series [3, 4, 5, 6, 7, 8, 9]. SSRIs with lower anticholinergic side effects, such as citalopram and escitalopram, are typically preferred [10].

Antipsychotics are also often used off‐label in FTD. However, their use needs close surveillance because of considerable risk of extrapyramidal side effects and the black box warning when treating dementia‐related behavioural symptoms in the elderly. Apart from the serotonin deficit, FTD is also associated with a dopaminergic deficit [11] and there is evidence that the mesolimbic and mesocortical dopaminergic pathway changes are related to the behavioural symptoms [12]. But still, several antipsychotics have demonstrated improvement in behavioural symptoms in FTD, including delusions or agitation, and in caregiver burden [13, 14, 15, 16, 17]. Because of the effect of antipsychotics on the nigrostriatal pathway, antipsychotics with lower D2‐receptor blocking affinity, such as quetiapine, are commonly preferred. A case series describing medication responses in FTD showed that quetiapine improved agitation in three patients [17].

Trazodone, a mixed agonist and antagonist of various serotonin receptors and antagonist of adrenergic receptors, is a third option often prescribed for neuropsychiatric symptoms in FTD. Trazodone increases extracellular serotonin in the frontal lobes and has been proved to decrease agitation and aggression and to improve sleep in FTD [18]. A randomized controlled trial with trazodone in FTD in a cohort of 26 cases showed a significant improvement in the Neuropsychiatric Inventory (NPI) total score, mainly based on improvements in irritability, agitation, depressive symptoms and eating disorders [19].

Other medications occasionally considered in behavioural manifestations of FTD include anticonvulsants, stimulants, benzodiazepines and other antidepressants. Acetylcholinesterase inhibitors, especially donepezil, frequently used to improve cognitive functioning in Alzheimer's disease, were proved to worsen the neuropsychiatric symptoms without cognitive improvement in patients with FTD in multiple studies [2, 18, 20, 21, 22, 23]. Memantine is also not an effective treatment for FTD [24, 25, 26, 27, 28].

Altogether, trial‐based evidence for symptomatic pharmacological treatment of behavioural disturbances in FTD is scarce despite their significant burden on both patients and caregivers. This expert opinion review aims to provide guidance for pharmacological treatment of behavioural symptoms that severely impact the patient's and family's wellbeing.

The symptoms queried were selected based on clinical expertise of the FTD disease group. They were deliberately meant to be concrete and directly taken from clinical experience rather than querying more general classes of symptom clusters.

## METHOD

This study is an expert opinion review based on the current practices within the 29 specialized centres of the FTD disease group of the European Reference Network for Rare Neurological Diseases (ERN‐RND). ERN‐RND was established in 2017 as one of the 24 European Reference Networks by the European Board of Member States and has currently 71 members from 24 EU countries. ERN‐RND aims to improve the healthcare of rare disease patients in the EU and to reduce inequalities in how healthcare is being provided for rare disease patients.

Neurologists or psychiatrists, who are faculty members at each participating ERN‐RND site and are clinically involved in the FTD clinical programme, were invited to participate in a survey. The primary objective was to evaluate current clinical practices concerning drug management for behavioural manifestations of FTD at their respective sites. The study encompassed 14 common behavioural problems and, for each of them, respondents were presented with a list of 20 drug options. This list also included ‘none’ and ‘other’ to allow physicians to specify if the preferred drug was not on the provided list.

The selected 14 behavioural problems in this survey consisted of physical aggression, verbal aggression, obsessive delusions, impulsivity, nightly unrest, self‐harm due to obsessive motor behaviour, sexual disinhibition, motor unrest, intentional self‐injury, apathy, hyperphagia, perseverative somatic complaints, rigidity of thought and loss of empathy. The choice of these behavioural disturbances, grounded in common clinical complaints, was determined by consensus by the leading study physicians (RV, HS, DS, RR). Obsessive delusions are persistent repetitive delusions that focus on specific content over an extended period (months). Self‐harm due to obsessive motor behaviour refers to harmful consequences to the patient's physical integrity caused by obsessive motor behaviour, such as repetitive tapping or rubbing or obsessive cleaning leading to abrasures and superficial wounds. Intentional self‐injury refers to motor behaviours deliberately aimed at causing harm to the body, such as cutting out pigmented spots or cutting body parts with scissors. Perseverative somatic complaints are perseverative physical complaints for which no organic cause can be identified. Apathy denotes a lack of motivation reflected in decreased goal‐directed behaviours, cognitions and emotions. Nightly unrest is characterized by increased nocturnal activity and difficulty remaining in bed. Motor unrest describes restlessness and stereotypical movements. Prior to the survey, the participants were informed about the list of symptoms, and the above definitions, including the examples, were given for terms that may not have been clear from the start.

Most of the specific symptoms queried can be mapped onto one or more general classes from the different FTD symptom classification schemes (Table 1). According to the Rascovsky et al. (2011) consensus criteria [29], physical and verbal aggression, impulsivity and sexual disinhibition would probably be classified under behavioural disinhibition. Obsessive delusions, self‐harm due to obsessive motor behaviour, intentional self‐injury, perseverative somatic complaints and rigidity of thought would probably be classified under perseverative, stereotyped or compulsive/ritualistic behaviour. Apathy corresponds to apathy or inertia in the Rascovsky et al. (2011) classification, loss of empathy corresponds to loss of sympathy or empathy, and hyperphagia is mentioned under hyperorality and dietary changes in the Rascovsky et al. criteria. Nightly and motor unrest are more difficult to classify under one of the mentioned categories and can result from disinhibition, apathy with low daytime activity or from obsessive‐repetitive behaviours.

**TABLE 1:** Categories of different FTD symptom classification schemes wherein the queried behavioural symptoms would fall.

Behavioural symptom	Rascovsky et al. [29]	NPI‐Q	GenFi neuropsychiatric clinical questionnaire [30]
Physical aggression	Behavioural disinhibition	Agitation/aggression	Agitation/aggression
Verbal aggression	Behavioural disinhibition	Agitation/aggression	Agitation/aggression
Obsessive delusions	Obsessive‐repetitive behaviour	Delusions	Delusions/hallucinations
Impulsivity	Behavioural disinhibition	Disinhibition	Irritability/lability
Nightly unrest		Night‐time behavioural disturbances	Impaired sleep
Self‐harm due to obsessive motor behaviour	Obsessive‐repetitive behaviour	Aberrant motor behaviour	Aberrant motor behaviour
Sexual disinhibition	Behavioural disinhibition	Disinhibition	Hypersexuality
Motor unrest		Agitation/aggression	Aberrant motor behaviour
Intentional self‐injury	Obsessive‐repetitive behaviour		Aberrant motor behaviour
Apathy	Apathy	Apathy/indifference	
Hyperphagia	Hyperorality and dietary changes	Appetite/eating disturbance	
Perseverative somatic complaints	Obsessive‐repetitive behaviour	Anxiety, dysphoria	
Rigidity of thought	Obsessive‐repetitive behaviour		
Loss of empathy	Loss of sympathy and empathy	Apathy/indifference	

*Note*: The Rascovsky criteria form the basis for the behavioural module of the CDR plus NACC FTLD rating.

Abbreviations: CDR plus NACC FTLD, Clinical Dementia Rating plus National Alzheimer's Coordinating Centre Frontotemporal Lobar Degeneration; GenFi, Genetic Frontotemporal Dementia Initiative; NPI‐Q, Neuropsychiatric Inventory Questionnaire.

The 18 drug options included trazodone, sodium valproate, sertraline, semaglutide, risperidone, quetiapine, promazine, periciazine, oxazepam, olanzapine, mirtazapine, methylphenidate, hydroxyzine, fluoxetine, carbamazepine, bupropion, amitriptyline and (es)citalopram, plus ‘none’ and ‘other’. The choice of drugs was grounded on clinical practices, previous studies and theoretical mechanisms of action, as described above, and was also determined by consensus by the leading study physicians.

Participating physicians were instructed to respond according to their actual clinical practice. They were first asked about the availability of each mentioned drug in their respective countries. Subsequently, for each of the 14 behavioural disturbances:

1. Participants were asked to indicate by ticking a box if none of the suggested drug options was recommended. Alternatively, they were prompted to rank their top three recommended drug treatments with the instruction, ‘Please choose from the list of the following drug therapies the three most highly recommended’. An option ‘other’ was provided to allow physicians to specify any additional drugs they might recommend beyond the given list.
2. Similarly, they were asked to tick a box if none of the drug options was advised against. Alternatively, physicians could rank the three drug treatment options they considered strongly contraindicated with the instruction, ‘Please choose from the list of the following drug therapies the three certainly not to be used’.

It is worth noting that participating physicians were also questioned about nonpharmacological treatments. However, in order to maintain conciseness, the decision was made to exclude this information from the final version of this article.

### Statistical analysis

Two key indicators were computed to summarize the most recommended and the most contraindicated treatments for each behavioural disturbance: (1) the percentage of respondents amongst the participating physicians who selected a treatment regardless of its rank and (2) a weighted score (WS) that considered the rank. The WS was calculated by considering the average rank or mean score based on the physicians' ranking. Specifically, the first choice was assigned 3 points, the second choice 2 points, the third choice 1 point, and subsequent choices, if any, received 0.5 points, with no points awarded if not chosen. To estimate 95% credibility intervals for both indicators concerning each behavioural disturbance and treatment, 1000 bootstrapped samples were run for each statistical analysis.

To identify symptoms for which physicians recommended or advised against similar treatments, two principal component analyses (PCA) were performed, one focusing on recommended treatments and the other on contraindicated treatments. Each PCA was based on the percentage of physicians who selected a treatment, regardless of its rank. The dataset used for the analyses consisted of treatments as observations and behavioural disturbances as variables. This dataset structure allowed the exploration of patterns in treatment recommendations and contraindications across various behavioural symptoms.

## RESULTS

### Recommended treatments

**FIGURE 1:**
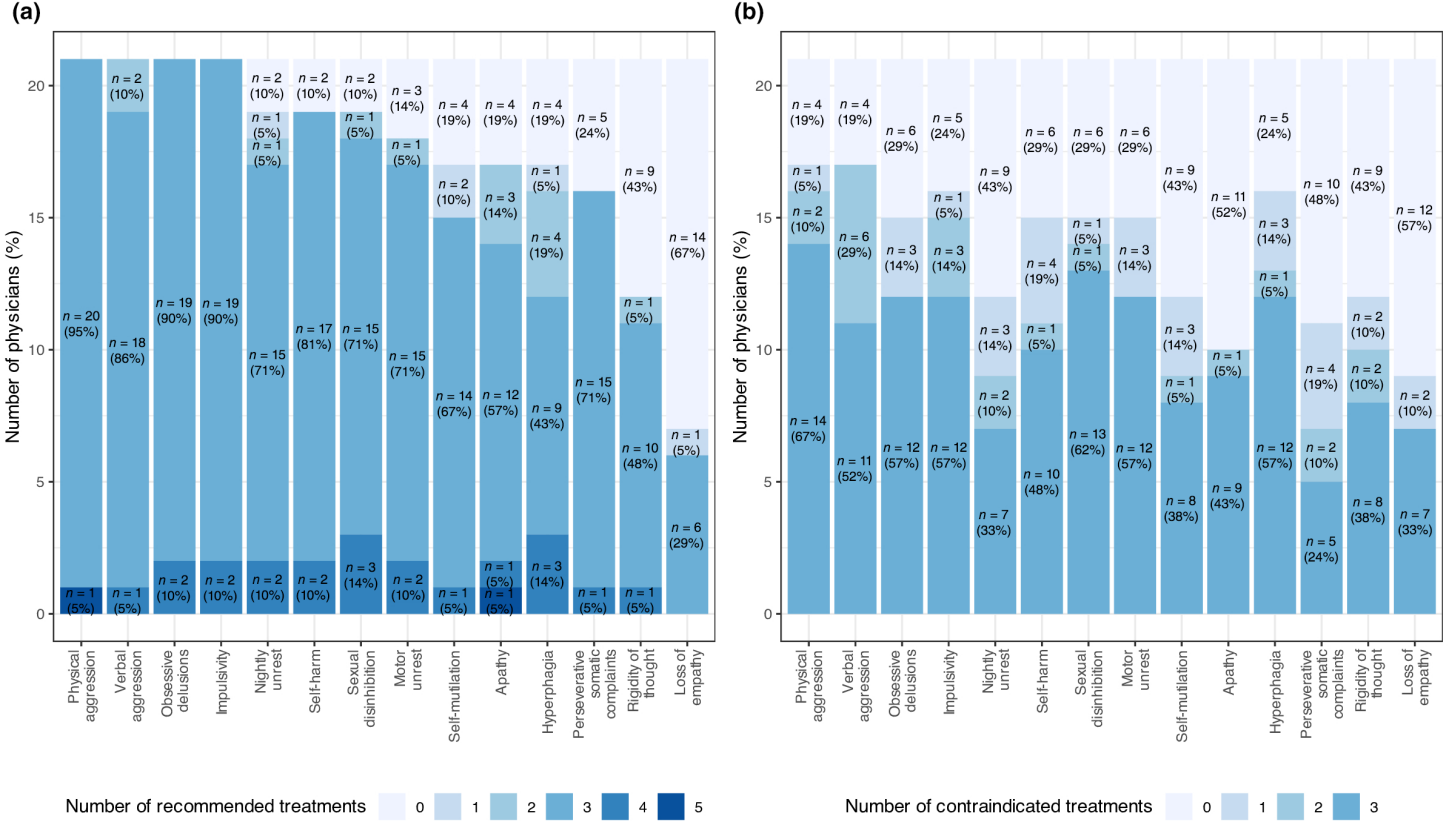
Distribution of physicians by the number of treatments selected for each behavioural symptom recommended by physicians (a) or marked as contraindicated (b). The symptoms are ordered along the *x*‐axis, with those having the highest number of physicians not selecting any recommended treatment on the right, whilst symptoms where all physicians chose at least one treatment are positioned on the left. The same ordering was applied in the contraindication figure. For example, in the case of loss of empathy: amongst the 21 physicians 14 (67%) did not recommended any treatment, one (5%) recommended a single treatment and six (29%) recommended three treatments.

Twenty‐one respondents from 19 centres across 13 countries participated. Depending on the symptoms, physicians exhibited varying degrees of willingness to prioritize treatments, as displayed in Figures 1a and S1. Notably, participating physicians were most comfortable with ranking recommended treatments in the case of physical aggression (100% ordered at least three treatments, as requested in the instructions), verbal aggression (90.5% ordered at least three treatments, and 9.5% recommended two treatments), obsessive delusions (100% at least three) and impulsivity (100% also ordering at least three).

In contrast, when addressing loss of empathy, 66.7% of the physicians (14 out of 21) opted not to recommend any proposed drug. Similarly, for rigidity of thought, 42.9% of physicians refrained from proposing or choosing any of the suggested treatments.

In several behavioural disturbances such as physical aggression, verbal aggression, perseverative somatic complaints or rigidity of thought, a clear consensus emerged with physicians favouring one, two or three treatments. However, for other conditions such as sexual disinhibition, self‐injury or hyperphagia, no such consensus was reached.

**FIGURE 2:**
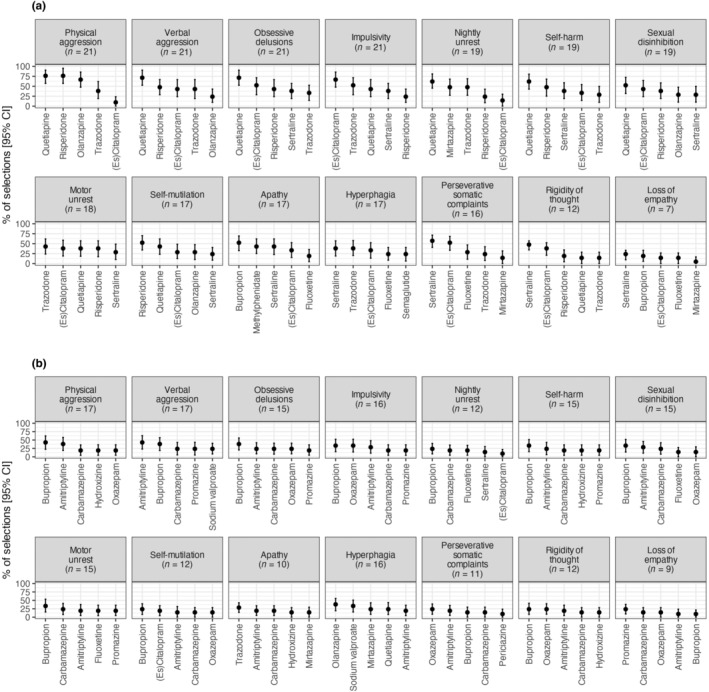
The top five most selected treatments by physicians for each behavioural symptom recommended by physicians (a) or marked as contraindicated (b). This figure shows, for each behavioural symptom and drug, the percentages of physicians (out of 21) who selected the drug, regardless of the rank, with their 95% bootstrapped confidence intervals. The *n* value in parentheses for each symptom represents the number of physicians who selected at least one treatment, giving insight into the sample size contributing to the calculations.

**FIGURE 3:**
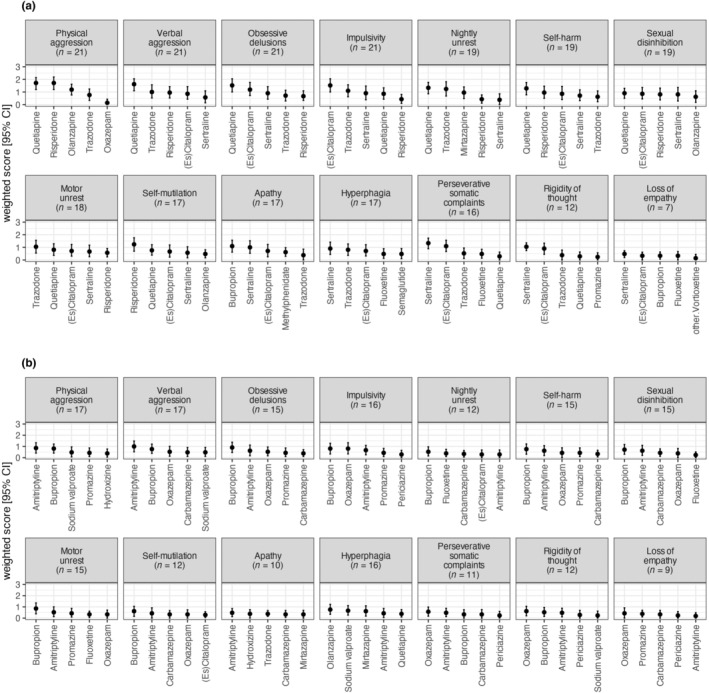
The top five treatments with highest mean score for each behavioural symptom recommended by physicians (a) or marked as contraindicated (b). This figure portrays the mean score allocated to a treatment per behavioural symptom. The scoring system is structured as follows: the first choice is awarded 3 points, the second choice receives 2 points, the third choice is given 1 point, subsequent choices, if any, get 0.5 points each, and, if the physician did not choose any treatment, 0 points are assigned. The *n* value in parentheses for each symptom represents the number of physicians who selected at least one treatment, providing context about the sample size contributing to the mean scores.

Amongst the behavioural disturbances, antipsychotics emerged as the most recommended for half of the symptoms (7/14), whilst SSRIs were the primary choice for 36% (5/14) of the symptoms (Figures 2a and 3a). Within the antipsychotic category, quetiapine was numerically the most selected treatment for six behavioural symptoms, including physical aggression (76.2% of all participating physicians, with a WS of 1.7 for both quetiapine and risperidone), obsessive delusions and verbal aggression (71.4% each; WS = 1.5 and WS = 1.6 respectively), nightly unrest and self‐harm due to obsessive motor behaviour (61.9% each, WS = 1.3) and sexual disinhibition (52.4%, WS = 0.9). Risperidone was the preferred choice for self‐injury (52.4%, WS = 1.2).

Within the SSRIs, sertraline was the most selected treatment for four behavioural symptoms: perseverative somatic complaints (57.1%, WS = 1.3), rigidity of thought (47.6%, WS = 1.0), hyperphagia (38.1%, WS = 0.9) and loss of empathy (23.8%, WS = 0.5). (Es)citalopram was the most selected for impulsivity (66.7%, WS = 1.5). For motor unrest, trazodone was the preferred treatment (42.9%, WS = 1.0), whilst bupropion was favoured for apathy (52.4%, WS = 1.1).

Concluding, at least two‐thirds of physicians selected at least one identical treatment for the four following behavioural symptoms: physical aggression, verbal aggression, obsessive delusions and impulsivity. These were also the four symptoms with a mean rank (i.e., WS) higher than 1.5, namely for two antipsychotics, emphasizing the consistency in physician preferences for these specific symptoms.

**FIGURE 4:**
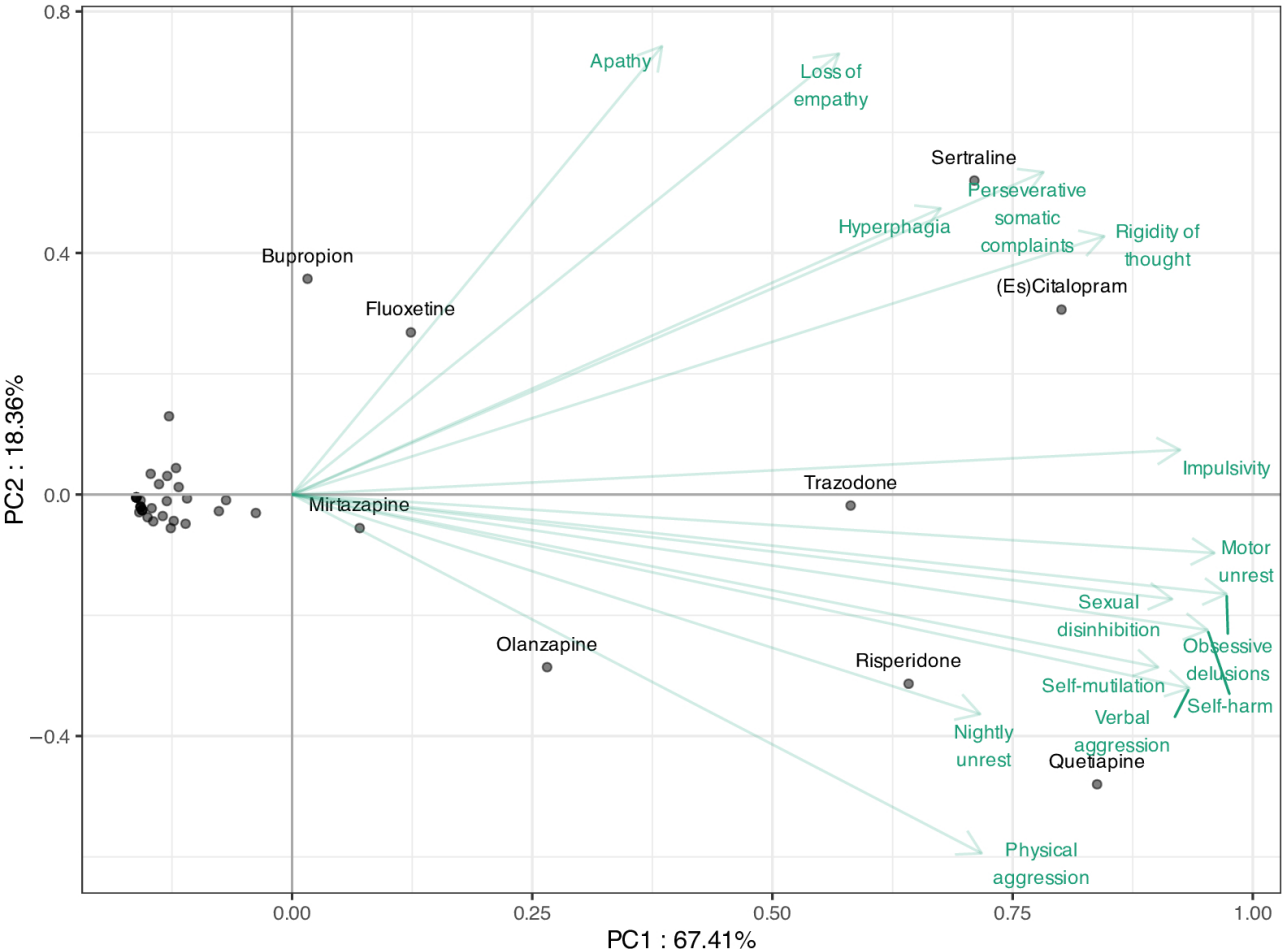
Results of the PCA based on the percentage of physicians who selected a treatment, regardless of its rank, using the treatments as observations and the behavioural disturbances as variables. PCA, principal components analysis.

Figure 4 shows the PCA. The first component of the PCA captures 67.4% of the dataset variance, whilst the second dimension accounts for 18.4%. The first dimension primarily represents a size effect, with all symptom coefficients being positive: treatments located further to the right on the figure exhibit higher citation counts. Conversely, the second dimension distinguishes between treatments with similar citation patterns based on difference in target symptoms.

Based on the PCA, sertraline and (es)citalopram are close and thus similarly recommended for hyperphagia, perseverative somatic complaints and rigidity of thought, whereas they are rarely suggested for physical aggression and nightly unrest. These two treatments are also endorsed for apathy and loss of empathy, alongside bupropion and fluoxetine. Quetiapine and risperidone are frequently co‐cited, particularly for nightly unrest and physical aggression (with olanzapine for physical aggression), but are seldom mentioned for hyperphagia, perseverative somatic complaints and rigidity of thought. For other symptoms, although quetiapine and risperidone are the most frequently cited, sertraline and (es)citalopram are also commonly recommended.

However, clustering is not able to encompass the remaining behavioural symptoms, i.e. impulsivity, motor unrest, sexual disinhibition, apathy and obsessive delusions. For these five symptoms either different drug classes or both SSRIs and antipsychotics are advised.

### Contraindicated treatments

The task of selecting contraindicated treatments proved more challenging for physicians compared to making recommendations (Figure 1b). For all behavioural symptoms there are at least four (19%) physicians who did not select any treatment, emphasizing the complexity and hesitancy in identifying contraindicated options.

There was also a varying response rate across symptoms. Physical aggression and verbal aggression had the highest response rates (81% of physicians selected at least one treatment), whilst loss of empathy had the lowest response rate (43%).

Furthermore, the results varied when considering the percentage of physicians who selected a treatment regardless of its rank (Figure 2b) and the WS that considered the rank (Figure 3b).

Considering the percentage regardless of rank, bupropion was selected as most contraindicated in nine behavioural symptoms: physical aggression (selected by 42.8% of physicians), obsessive delusions (38.1%), impulsivity, self‐harm, sexual disinhibition and motor unrest (33.4% each), nightly unrest, self‐injury and rigidity of thought (23.8% each). Amitriptyline was identified as the most contraindicated for verbal aggression (42.8%), olanzapine for hyperphagia (38.1%), oxazepam for perseverative somatic complaints (23.8%), promazine for loss of empathy (23.8%) and trazodone for apathy (28.6%).

Considering the WS for contraindicated treatments, amitriptyline was the most contraindicated in three symptoms including verbal aggression (WS = 1), physical aggression (WS = 0.9) and apathy (WS = 0.5). Bupropion was the most selected as contraindicated in seven behavioural symptoms: obsessive delusions and motor unrest (WS = 0.9 each), impulsivity and self‐harm (WS = 0.8 each), sexual disinhibition (WS = 0.7), self‐injury (WS = 0.6) and nightly unrest (WS = 0.5). Oxazepam was the most contraindicated for rigidity of thought (WS = 0.6), perseverative somatic complaints (WS = 0.6) and loss of empathy (WS = 0.4). Olanzapine was the most contraindicated for hyperphagia (WS = 0.8). These results showed that there is no clear consensus between the 21 physicians concerning the contraindicated treatments, which is probably influenced by individual clinical experiences, patient profiles and varying interpretations of contraindications for specific behavioural symptoms.

## DISCUSSION

Our study looked at the pharmacological preferences of neurologists and psychiatrists, all members of the ERN‐RND network, with expertise in cognitive disorders for common behavioural symptoms in FTD. The main findings are as follows: (i) there was a strong consensus for drug therapy in four specific behavioural manifestations (verbal aggression, physical aggression, obsessive delusions and impulsivity); (ii) therapeutic options for other behavioural symptoms were more heterogeneous; and (iii) either SSRIs or antipsychotics are most often advised depending on the target symptom.

Our results indicated a strong consensus amongst participating physicians that drug therapy was warranted for four specific behavioural disturbances: verbal aggression, physical aggression, impulsivity and obsessive delusions. It can be hypothesized that the reason for this common viewpoint across all ERN‐RND centres is that these symptoms both represent an important burden for the patient and/or caregiver and tend to respond favourably to pharmacological treatment.

The PCA in this study indicates distinctive patterns in pharmacological preferences for behavioural symptoms in FTD based on therapeutic preferences for SSRI versus antipsychotics. This classification was an interesting *post hoc* finding as a result of statistical data analysis and seems to reflect not only therapeutic habitudes but probably also underlying expert experience. One group, encompassing perseverative somatic complaints, rigidity of thought, hyperphagia and loss of empathy, are preferentially treated with SSRIs. These manifestations may result from emotional disturbances, interrupted orbitofrontal and dorsolateral prefrontal circuits and serotonergic deficits [11, 31, 32]. Conversely, antipsychotics are preferred for another group of symptoms, including physical aggression, verbal aggression, self‐injury, self‐harm due to obsessive motor behaviour, and nightly unrest. These manifestations may reflect loss of self‐control, aberrant motor behaviour and auto/hetero‐aggressivity and relate to cortico‐subcortical circuits, mediofrontal areas and noradrenaline and dopamine alterations [31, 32].

The common use of SSRIs aligns the known presynaptic serotonin deficits and loss of cortical serotoninergic innervation in FTD [2]. The preference for quetiapine may stem from its classification as a second‐generation neuroleptic with low affinity to the D2 receptor.

Principal component analysis is a standard statistical way to detect the latent structure in the data. The data are composed of the response options provided by the experts who participated. The fact that symptoms can be grouped based on similar drug treatment decisions does not contradict the importance of individually tailored management of symptoms. It indicates that the individually tailored management happens in a relatively consistent way across different centres.

Furthermore, trazodone was a top five choice in 10 out of 14 behavioural symptoms and the first choice for motor unrest, mirtazapine was the second choice for nightly unrest and methylphenidate and bupropion were ranked highly for apathy. Semaglutide was advised for hyperphagia by one in four physicians. Drugs that never made it into the top five advised medications were sodium valproate, periciazine, hydroxyzine, carbamazepine and amitriptyline.

Additionally, bupropion was strongly advised against in 10 out of the 14 behavioural symptoms, emphasizing a clear recommendation against its use in the majority of cases.

### Study strengths

In this study, there was a large participation rate consisting of 21 physicians from the FTD group of the ERN‐RND, specialized in the regular treatment of patients with FTD. This expert review represents a pioneering effort in the field, providing insights into recommended treatments for individuals with FTD. As the first of its kind, this study holds significant importance in advancing our understanding of FTD management from real‐life data. The findings from this research have the potential to serve as a valuable resource, guiding the selection of future drugs and informing the design of forthcoming clinical trials aimed at enhancing FTD treatment strategies.

### Study limitations

The description of current practices in expert centres should be viewed with caution, as it does not serve as proof of efficacy. Whilst these practices provide valuable insights into the real‐world application of treatments, they do not necessarily establish their effectiveness. It is important to recognize that relying solely on clinical experience for defining target symptoms might vary, as different experts may prioritize symptoms differently. Certain specific symptoms, such as loss of manners, and bothersome symptoms like depression and anxiety were not queried in this study. Furthermore, the behavioural symptoms queried were concrete and directly taken from clinical experience rather than more general classes of symptom clusters. Without using a standardized set of symptoms, there is a risk of subjectivity in identifying and addressing target symptoms, highlighting the need for more rigorous and objective criteria in the evaluation and development of treatment approaches. Finally, nonpharmacological measures were also evaluated in the study; however, to maintain conciseness, these specific data were excluded from the final version.

## CONCLUSIONS

This study reveals several insights regarding the treatment preferences for behavioural symptoms associated with FTD. The highest consensus for treatment was observed for physical and verbal aggression, impulsivity and obsessive delusions. This suggests a more unified approach amongst physicians in addressing these specific behavioural challenges associated with FTD. PCA suggests a distinction between a group which are best treated with SSRIs and a group for which antipsychotics are considered more effective.

Furthermore, trazodone was a top five choice in 10 out of 14 behavioural symptoms, mirtazapine was the second choice for nightly unrest and methylphenidate and bupropion were ranked highly for apathy. Semaglutide was advised for hyperphagia by one in four physicians. Drugs that never made it into the top five advised medications were sodium valproate, periciazine, hydroxyzine, carbamazepine and amitriptyline. Bupropion was strongly advised against in 10 out of the 14 behavioural symptoms, emphasizing a clear recommendation against its use in the majority of cases.

The survey data offer insights that may inform prioritization and design of therapeutic studies, particularly for existing drugs targeting behavioural disturbances in FTD. Additionally, the survey data can provide expert guidance that is helpful for healthcare professionals involved in the treatment of behavioural symptoms impacting the wellbeing of both patients and their families. This expertise can aid in developing more tailored and effective therapeutic approaches for managing FTD‐associated behaviours.

## AUTHOR CONTRIBUTIONS

**Casper Wittebrood:** Writing – original draft; writing – review and editing; formal analysis; methodology; visualization; conceptualization; data curation. **Marina Boban:** Writing – review and editing; investigation. **Annchiara Cagnin:** Investigation; writing – review and editing. **Sabina Capellari:** Investigation; writing – review and editing. **François‐Laurent De Winter:** Investigation; writing – review and editing. **Atbin Djamshidian:** Investigation; writing – review and editing. **Manuel Menéndez González:** Investigation; writing – review and editing. **Lena E. Hjermind:** Investigation; writing – review and editing. **Lenka Krajcovicova:** Investigation; writing – review and editing. **Johanna Krüger:** Investigation; writing – review and editing. **Johannes Levin:** Investigation; writing – review and editing. **Kathrin Reetz:** Investigation; writing – review and editing. **Eloy Rodriguez Rodriguez:** Investigation; writing – review and editing. **JD Rohrer:** Investigation; writing – review and editing. **Tim Van Langenhove:** Investigation; writing – review and editing. **Carola Reinhard:** Investigation; writing – review and editing. **Holm Graessner:** Investigation; writing – review and editing. **Robert Rusina:** Investigation; writing – review and editing. **Dario Saracino:** Investigation; writing – review and editing; data curation; supervision; methodology. **Marion Houot:** Visualization; formal analysis. **Harro Seelar:** Investigation; writing – review and editing. **Rik Vandenberghe:** Funding acquisition; investigation; conceptualization; validation; writing – review and editing; supervision.

## CONFLICT OF INTEREST STATEMENT

Johannes Levin reports receiving speaker fees from Bayer Vital, Biogen, EISAI, TEVA, Zambon, Merck, and Roche, as well as consulting fees from Axon Neuroscience, EISAI, and Biogen. He has also received author fees from Thieme Medical Publishers and W. Kohlhammer GmbH Medical Publishers. Johannes Levin is the inventor of a patent titled "Oral Phenylbutyrate for Treatment of Human 4‐Repeat Tauopathies" (EP 23 156 122.6) filed by LMU Munich. Additionally, he serves as the Chief Medical Officer for MODAG GmbH, is a beneficiary of MODAG GmbH's phantom share program, and is the inventor of a patent titled "Pharmaceutical Composition and Methods of Use" (EP 22 159 408.8) filed by MODAG GmbH. All of these activities are unrelated to the work submitted. Jonathan Rohrer has provided consultancy services or served on advisory boards for Novartis, Wave Life Sciences, Prevail, Alector, Aviado Bio, Takeda, Arkuda Therapeutics, and Denali Therapeutics.

## Supporting information


**Figure S1.** All treatments with a mean score for each behavioural symptom recommended by physicians.

## Data Availability

The data that support the findings of this study are available from the corresponding author upon reasonable request.
