# Correction to “Pharmacotherapy for behavioural manifestations in frontotemporal dementia: An expert consensus from the European Reference Network for Rare Neurological Diseases (ERN‐RND)”

**DOI:** 10.1111/ene.70108

**Published:** 2025-03-06

Wittebrood C , Boban M , Cagnin A , et al. Pharmacotherapy for behavioural manifestations in frontotemporal dementia: An expert consensus from the European Reference Network for Rare Neurological Diseases (ERN‐RND). Eur J Neurol. 2024; 31:e16446. 10.1111/ene.16446 39447217 PMC11555005

In the above article, an author's name was incorrectly published as Harro Seelar. It should have been Harro Seelaar.

The authors apologize for this error.

