# Supplementary figures and images for: Pharmacotherapy for behavioural manifestations in frontotemporal dementia: An expert consensus from the European Reference Network for Rare Neurological Diseases (ERN‐RND)
Source: Eur J Neurol. 2024 Oct 24;31(12):e16446. doi: 10.1111/ene.16446 (PMC11555005; doi:10.1111/ene.16446)

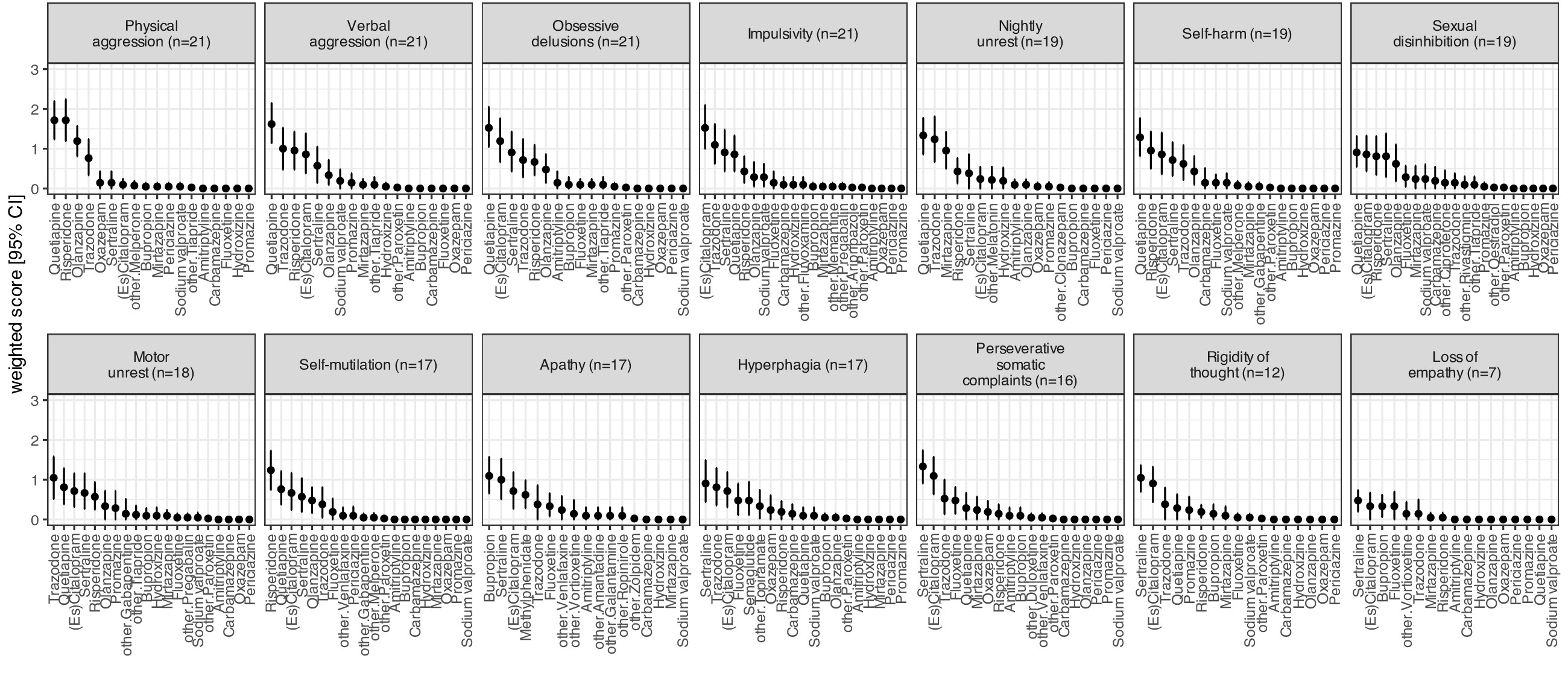

Supplement: Supplementary file 1 — Figure S1. All treatments with a mean score for each behavioural symptom recommended by physicians. [file ENE-31-e16446-s001.jpg]
